# Water management in anion-exchange membrane water electrolyzers under dry cathode operation†

**DOI:** 10.1039/d2ra03846c

**Published:** 2022-07-20

**Authors:** Susanne Koch, Joey Disch, Sophia K. Kilian, Yiyong Han, Lukas Metzler, Alessandro Tengattini, Lukas Helfen, Michael Schulz, Matthias Breitwieser, Severin Vierrath

**Affiliations:** Hahn-Schickard Georges-Koehler-Allee 103 79110 Freiburg Germany Severin.Vierrath@imtek.uni-freiburg.de; Electrochemical Energy Systems, IMTEK – Department of Microsystems Engineering, University of Freiburg Georges-Koehler-Allee 103 79110 Freiburg Germany; FIT, University of Freiburg Georges-Koehler-Allee 105 79110 Freiburg Germany; Heinz Maier-Leibnitz Center, Technical University Munich Garching Germany; Grenoble INP, CNRS, 3SR, Univ.Grenoble Alpes 38000 Grenoble France; Institut Laue-Langevin (ILL) 71 Avenue des Martyrs 38000 Grenoble France

## Abstract

Dry cathode operation is a desired operation mode in anion-exchange membrane water electrolyzers to minimize contamination of the generated hydrogen. However, water management under such operation conditions makes it challenging to maintain reliable performance and durability. Here, we utilize high-resolution *in situ* neutron imaging (∼6 μm effective resolution) to analyze the water content inside the membrane-electrode-assembly of an anion-exchange membrane water electrolyzer. The ion-exchange capacity (IEC) and thus hydrophilicity of the polymer binder in the cathode catalyst layer is varied to study the influence on water content in the anode (mid IEC, 1.8–2.2 meq. g^−1^ and high IEC, 2.3–2.6 meq. g^−1^). The neutron radiographies show that a higher ion-exchange capacity binder allows improved water retention, which reduces the drying-out of the cathode at high current densities. Electrochemical measurements confirm a generally better efficiency for a high IEC cell above 600 mA cm^−2^. At 1.5 A cm^−2^ the high IEC has a 100 mV lower overpotential (2.1 V *vs.* 2.2 V) and a lower high frequency resistance (210 mΩ cm^−2^*vs.* 255 mΩ cm^−2^), which is believed to be linked to the improved cathode water retention and membrane humidification. As a consequence, the performance stability of the high IEC cell at 1 A cm^−2^ is also significantly better than that of the mid IEC cell (45 mV h^−1^*vs.* 75 mV h^−1^).

## Abstract 2

Dry cathode operation is a desired operation mode in anion-exchange membrane water electrolyzers, but water management is crucial. This is visualized using high-resolution neutron radiography and the ion-exchange capacity of the cathode ionomer is varied.

## Graphical abstract

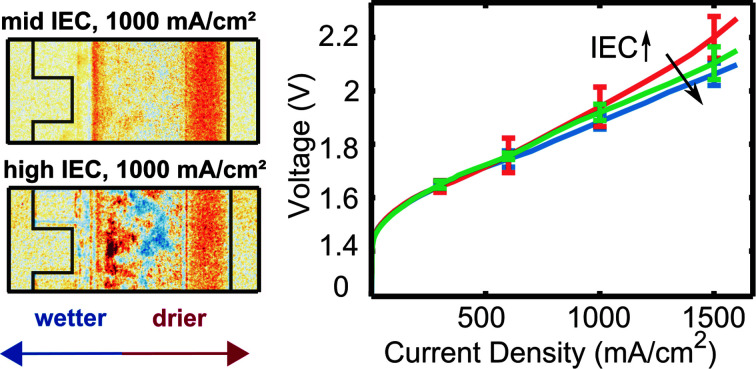

## Introduction

Green hydrogen *via* water electrolysis is a key to sustainable steel production, synthetic fuels and in general to decarbonize the chemical industry.^1,2^ To meet the future demand for green hydrogen, the cost and environmental footprint of water electrolyzers have to be reduced and the use of rare raw materials has to be minimized. While liquid-alkaline and proton-exchange membrane water electrolysis are more mature, anion-exchange membrane (AEM) water electrolysis offers the possibility to make use of the benefits of PEM water electrolyzers, such as operation at high current densities and differential pressure, whilst keeping the advantages of alkaline electrolysis, like using non-noble catalyst instead of iridium.^1,3,4^

While AEM electrolysis is operated with an anion-exchange membrane and typically both electrodes immersed in low molarity KOH, using a dry cathode without feed solution has proven a successful way to reduce contamination of the hydrogen product stream.^5–9^ However, the stability of the AEM and binder polymer and their capability to transport anions is directly linked to their water/KOH content and thus the overall performance and lifetime can strongly suffer from (local) dry-outs.^10,11^ Multiple studies investigated the use of a dry cathode^9,12^ highlighting the influence of different factors on performance and durability such as anode binder content,^8^ membrane type and thickness,^13^ and catalyst loading,^5^ but without analyzing the water distribution any further.

In this work, we study the water distribution and dry-out during dry cathode operation of a catalyst-coated membrane (CCM) using neutron imaging. In order to counter the dry-out of the cathode we increased the anion-exchange capacity (IEC) of the cathode binder and studied its effect on water retention in the catalyst layer and subsequent dry-out during operation at higher currents.

## Methods

### Materials

Anion-exchange membranes (AF2-HLE7-25-X, reinforced, 25 μm) and ionomer (AP2-HNN6-00-X, IEC = 1.8–2.2 meq. g^−1^, denoted as “mid IEC”, and AP2-HNN8-00-X, IEC = 2.3–2.6 meq. g^−1^, denoted as “high IEC”) were provided by Ionomr Innovations Inc. IrO_*x*_ powder (Premion, 99.99%, Alfa Aesar) and Pt/C (60 wt%, Greenerity) were used as anode and cathode catalysts, respectively. Ethanol (EtOH, 99.5% Ph. Ezr., extra pure) and 2-propanol (IPA, 70%, pure) were purchased from Carl Roth GmbH + Co. KG. Potassium hydroxide (KOH) pellets (85%, Sigma Aldrich) were used to prepare solutions for ion-exchange and the liquid electrolyte. Nickel fiber felts (Bekipor® 2NI30-1, 0, 1 mm thickness, 83% porosity) were purchased from Bekaert and Freudenberg H24C5 carbon paper with a microporous layer was purchased from FuelCellStore.

### CCM fabrication

Catalyst-coated membranes (CCMs) were fabricated and handled following the methods previously published.^14^ The membrane and ionomer shipped in mixed iodide-chloride form were exchanged to the OH^−^ form by immersion in 3 M KOH for 24 h followed by at least 24 h in 1 M KOH before assembly.^15^ Samples for neutron imaging were stored in 1 M KOH for a week for transport, the storage time in 1 M KOH on this scale was confirmed to have minimal influence on the sample performance for two identically fabricated CCMs.

### Electrochemical measurements

To obtain the polarization curves and to perform the 6 hours degradation tests the samples were assembled, measured and preconditioned as detailed previously^14^ in a 4 cm^2^ active area cell fixture utilizing parallel flow fields. All measurements were performed with 0.1 M KOH feed on the anode side. For the neutron imaging experiments, the cell was assembled in a fixture and the test bench optimized for neutron imaging published by Disch *et al.*^16^ with an active area of 2 cm^2^. The flow field on the cathode side has a serpentine pattern, with the channels in line with the neutron beam direction, while the anode flow field has a parallel-pattern with channels perpendicular to the beam direction. For *in operando* electrolysis imaging the cells were kept at 50 mA cm^−2^, 100 mA cm^−2^, 200 mA cm^−2^, 500 mA cm^−2^ and 1000 mA cm^−2^ for 15 minutes each, moving from the lowest to the highest current density in a stepwise pattern. The cell denoted as high IEC was measured for an additional ten minutes at each current density for further neutron imaging tests not discussed here. At the end of each measurement an electrochemical impedance spectroscopy (EIS) measurement was performed at that current density using an amplitude of 5% of the applied DC current in a frequency range of 500 kHz to 1 kHz. The high-frequency resistance was then determined from the zero-crossing in the Nyquist plot of the EIS data set.

### Neutron imaging

*In situ* neutron imaging experiments were performed at the cold neutron beamline “NeXT” at the Institut Laue-Langevin in Grenoble.^17,18^ To enhance the resolution in the horizontal direction and maintain sufficient flux density at the sample position the beam was collimated by a 5 mm wide and 30 mm tall slit, resulting in a collimation ratio of 2000 along the horizontal and 333 along the vertical direction. An intensified neutron microscope from the ANTARES beamline at Forschungs-Neutronenquelle Heinz Maier-Leibnitz (FRM II) was used to perform neutron imaging. A 5 μm thick 157-Gd_2_O_2_S scintillator was used to convert the neutron beam into visible light. The microscope was made of two infinity corrected lenses, with a Zeiss 55 mm f/1.4 lens as the objective lens and a Nikon 70–200 mm f/2.8 zoom lens as the tube lens, to achieve a magnification between 1.27 and 3.63. To amplify the scintillation light coming out of the light microscope, an 18 mm diameter Photonis single stack image intensifier with a 1 : 1 relay lens was put between the microscope and the camera. The camera is a Hamamatsu Fusion BT sCMOS camera with 2304 : 2304 pixels and a pixel size of 6.5 μm. The magnification of the microscope was set to be 3.63, resulting a system resolution of ∼6 μm and a max field of view of 5.7 × 5.7 mm. The exposure time for each neutron radiographic image was set to 10 s. Gamma spots noise was removed *via* a previous developed denoiser for each neutron radiographic image.^19^ 90 frames electrolyzer images, 90 frames open beam images and 30 frames camera dark images were taken and averaged for each experiment to calculate the normalized neutron radiographic transmission images. Intensity images were normalized using a dark image taken without the neutron beam and an open beam image without the cell. The intensity map was calculated as
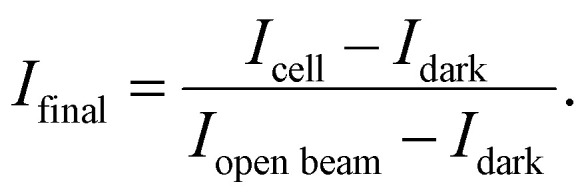


Because the AEMWE CCM needs to be assembled in its wet, hydroxide form and a drying of the materials significantly affects performance, no dry image of the cell could be acquired. Thus, no quantitative water content is determined from neutron images and only the relative water content in comparison between different current densities is analyzed in detail.

## Results & discussion

### *In situ* neutron imaging of anion-exchange water electrolyzers

**Fig. 1:**
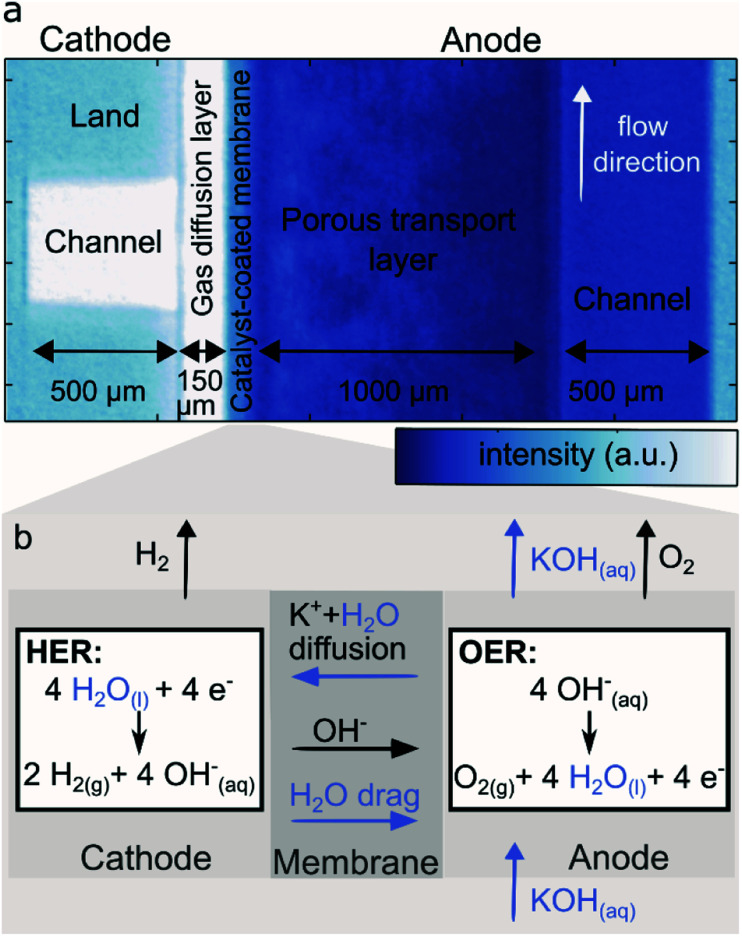
(a) Neutron image of one cathode flow channel of an AEM water electrolyzer, high neutron attenuation (blue) is an indicator of high water content. (b) Water transport processes and electrochemical reactions (hydrogen evolution reaction HER and oxygen evolution reaction OER) within an AEM water electrolyzer.

Fig. 1 depicts the neutron transmission image through the test cell, with one cathode land and channel on the left and flow direction perpendicular to the viewing plane. The right side features the anode where the flow direction is in-plane with the viewing pane from the bottom to the top of the image, thus representing the sum of all anode lands and channels. The cathode flow channel, parallel to the neutron beam direction is dry and thus shows high neutron transmission (white here), while the outline of the metal flow fields is visible due to the low, but non zero, absorption and scattering of the titanium bulk material and gold coating. The cathode transport layer (gas diffusion layer, GDL), made of carbon, is relatively dry, except for the edges, which may accumulate and retain some water in a thin droplet film. Despite the high resolution, the transition between catalyst layers and membrane is not clearly resolved in the images. However, an overall lower neutron transmission is observed for the CCM region in comparison to the GDL, which corresponds to a higher water content. The anode porous transport layer (PTL) shows a strong attenuation as it is soaked with aqueous KOH. The cathode GDL is compressed up to 55% of its original thickness. The anode PTL has a significantly higher thickness and rigidity, and is compressed only marginally. On the right side of the image, the anode flow channel is filled with aqueous KOH and thus appears significantly darker than the dry cathode flow channel and the metal ridges. The brightness difference to the anode PTL stems from the different materials.

Fig. 1b shows a schematic of the processes involving liquids (KOH, water) expected in an AEM water electrolyzer under dry cathode operation. Aqueous KOH and especially water are dragged across the membrane *via* diffusion due to the concentration gradient. During electrolysis the water is consumed at the cathode catalyst, evolving H_2_ which leaves though the gas channel. The cathode water is also dragged along to the anode within the solvation shell of OH^−^ migrating across the membrane (electro-osmotic drag). At the anode is water is generated in the oxygen evolution reaction. In summary, water is fed, generated and transported to the anode, while the cathode is only supplied with water *via* diffusion through the membrane. This strong imbalance can be clearly seen in Fig. 1a. Furthermore, a gradient in the anode PTL indicates evolved gaseous O_2_ reducing the water content closer to the membrane.

### Relative dry-out at high current densities

**Fig. 2:**
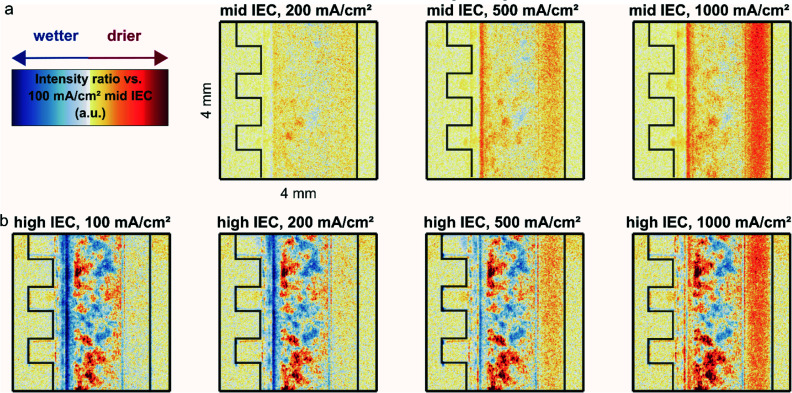
Relative drying of the AEM water electrolyzer fed with 0.1 M KOH only on the anode side with a mid IEC (1.8–2.2 meq. g^−1^) cathode (a) and a high IEC (2.3–2.6 meq. g^−1^) cathode (b) in comparison to the mid IEC cell at 100 mA cm^−2^. White denotes no change in neutron intensity, blue an increase in water content (lower intensity) and yellow to red a decrease in water content.

Fig. 2a shows the relative water content computed as the ratio between the normalized neutron image intensity at the indicated current density and the normalized image of the mid IEC (1.8–2.2 meq. g^−1^) cell at a reference current density of 100 mA cm^−2^. This reference point was chosen in order to avoid the slow system response at zero current operation. It was not possible to acquire a dry reference image, since the materials do not withstand dry assembly without significantly affecting performance.

At current densities higher than 100 mA cm^−2^ a significant drying of the anode flow channel is observed. During the application of a higher current, more water is split and more oxygen is produced, resulting in a gas–liquid mix with increasingly higher gas content in the anode flow channel. Across the anode flow channel there is also a decrease in water content from the bottom to the top of the image, which follows the direction of the liquid flow and natural travel path of the gas bubbles which rise towards the top of the cells parallel flow field before being transported out with the liquid.

The gradient inside the anode transport layer from the CCM towards the flow channel also becomes more apparent at higher current densities, which results from the same increased evolution of gases, indicated by an increasing red color. Close to the CCM a significant drying is observed for both cells for increasing current densities. The dryness is so significant it encompasses the area of the CCM as well as the edges of both the cathode and anode transport layers.

Fig. 2b shows the relative water content of the high IEC cell (2.3–2.6 meq. g^−1^). For a better comparison with the previous measurement it was again referenced to the 100 mA cm^−2^ operation of the mid IEC. To determine if the two cells are comparable, the regions where the materials are identical were studied. As presented in Fig. S1 (in ESI†) the intensity inside the metallic flow plates and the anode channel overlap closely. Thus, it is possible to compare both cells and draw conclusions on the effect of varying IEC of the cathode binder.

For 100 to 500 mA cm^−2^, Fig. 2b shows clearly that the CCM region of the cell with high IEC cathode binder has a higher water content compared to the cell with mid IEC cathode. The apparent heterogeneous wet and dry spots in the anode transport layer of the high IEC cell are consistent across various current densities and thus most likely stem from variations in the microstructure of the utilized nickel fiber felts, leading to a seemingly heterogeneous water distribution.

**Fig. 3:**
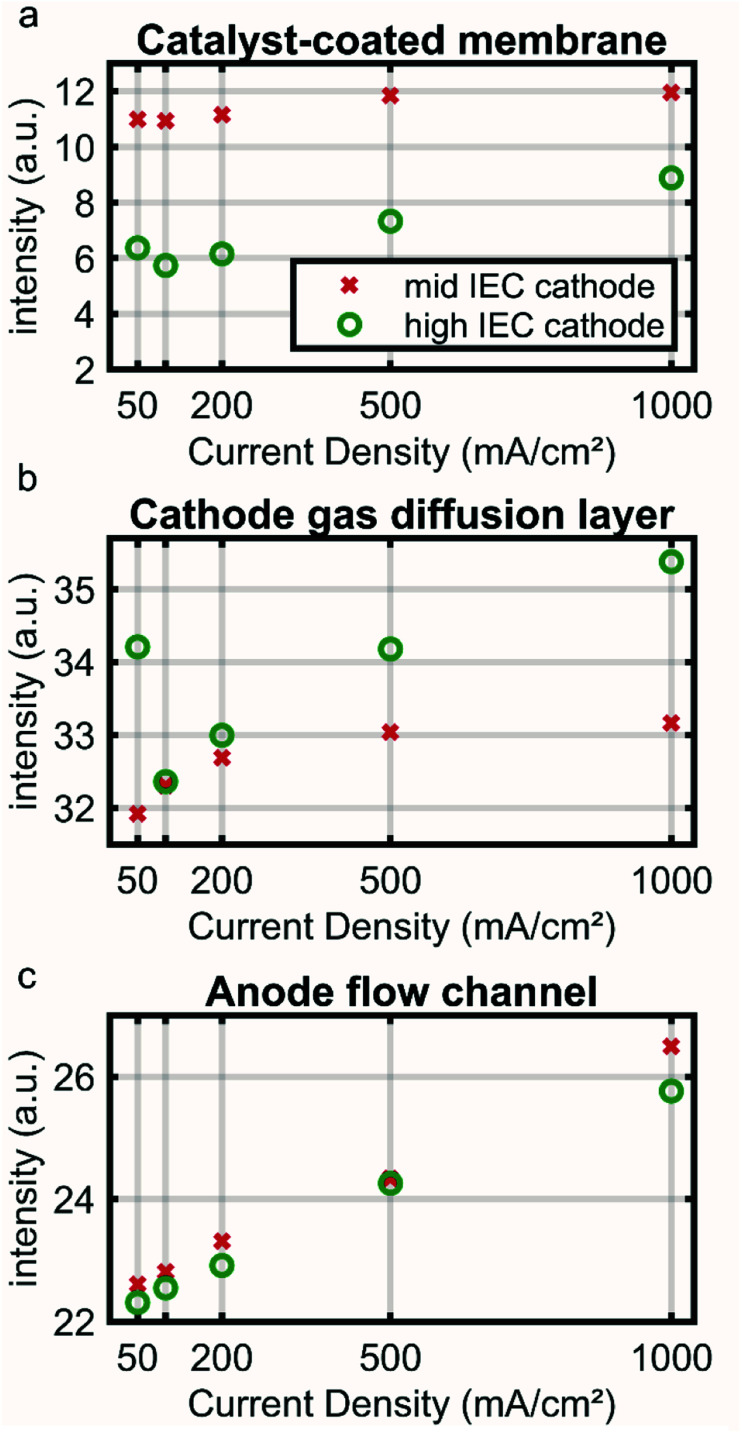
Variation in average dryness extracted from neutron images at different current densities in the regions corresponding to the cathode gas diffusion layer, membrane electrode assembly and anode flow channel. High intensity reflects low water content.

To illustrate this point further Fig. 3 shows an average intensity for the CCM (a), cathode GDL (b) and anode channel (c) regions (marked in Fig. S1a†) over the current density. Low neutron image intensity again correlates to a higher water content. The CCM region of the high IEC cell shows a lower intensity and thus higher water content than the mid IEC cell for all current densities. At 500 and 1000 mA cm^−2^ an increase in intensity indicates drying out of both CCMs.

In the CCM region the higher water content for the high IEC cell corresponds well to the current understanding. By attracting a hydration shell, the ionic groups within the polymeric binder are the main driving force for the water uptake. Thus, a higher IEC usually leads to a higher overall water content. However, with increasing current density a significant amount of water is removed by the reaction and electro-osmotic drag. This lowers the humidity in the gas phase and thus also the water hydration shell of the ionic groups.^20^ As a consequence a drying out at higher current densities is observed for both IECs.

The cathode GDL regions of the two cells show a similar water content at around 100 mA cm^−2^ and a decrease in water content (increase in intensity) for subsequent current densities (Fig. 3a). The 50 mA cm^−2^ likely has a larger error, because at this low current density the response of the cell is slow and the system may not be fully equilibrated within the given time (see Fig. S2†). The catalyst layer with higher IEC ionomer contains more water, indicated by the lower intensity in the neutron image (Fig. 3a green). Nevertheless, the high IEC cathode GDL dries out more at higher current densities (Fig. 3b – green). This apparent contradiction could be explained by the fact that the water is bound to more ionic groups in the catalyst layer and is not free to be released to the hydrophobic GDL.

The anode channel region shows a steady reduction in water content linearly correlated to the applied current (*R*^2^ ≈ 0.99). This linear correlation supports the hypothesis that the neutron signal in the anode is directly influenced by the evolving gas, which is in linear correlation to the applied current.

### Electrochemical performance

**Fig. 4:**
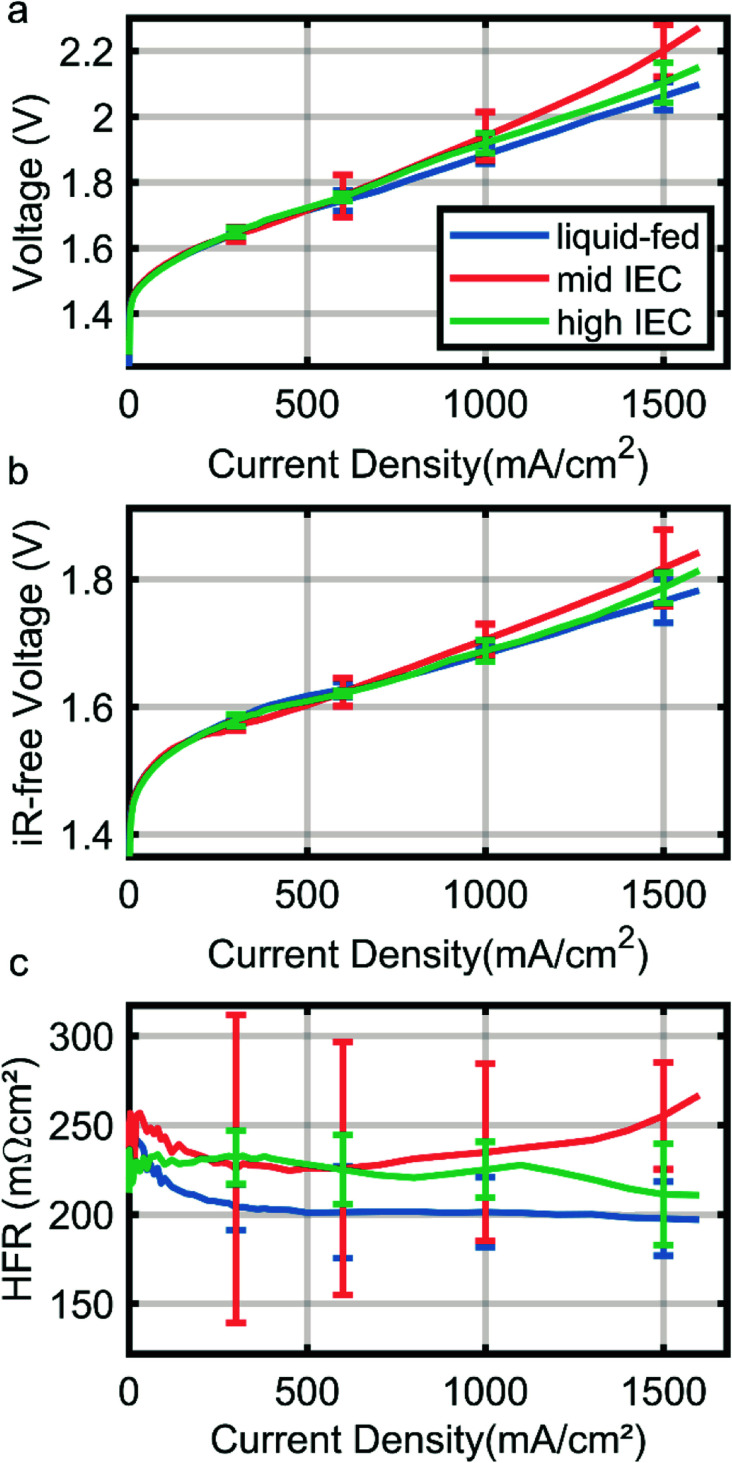
Polarization curve (a), *iR*-free voltage (b) and high frequency resistance extracted from electrochemical impedance spectroscopy (c) for 0.1 M KOH-fed cathode operation (blue) and dry cathode operation with mid IEC (orange, 1.8–2.2 meq. g^−1^) and high IEC (green, 2.3–2.6 meq. g^−1^) polymer in the cathode catalyst layer. Curves are the median for of three cells of each variation and the error bars denote the range in which the other samples results fall.

Fig. 4 shows the polarization curve of a liquid-fed cathode in comparison with two cells with a dry cathode (and 0.1 M KOH on the anode side). Curves are the median of three identically fabricated CCMs for each variation with the error bars denoting the range of the results of the three cells. The wet cathode operated CCM employs mid IEC binder in the cathode catalyst layer. All variations achieve 1 A cm^−2^ under 2 V (Fig. 4a), which is a current standard in AEMWE operation.^4^

While all variations overlap within the error bars, results indicate a trend above current densities of 600 mA cm^−2^. The liquid-fed cathode outperforms both dry cathode cells and also has a generally lower HFR (Fig. 4c). The HFR majorly reflects the ionic resistance of the membrane, which is thus lower due to a better membrane humidification compared to cells with the dry cathodes. While the neutron imaging results imply a much stronger drying out effect for dry cathode operation, these small differences in HFR are in line with recent reports by Kiessling *et al.* for the change from wet to dry operation on the cathode side.^12^ They also observe a small increase in HFR at high current densities only, likely due to the dehydration we observed in neutron imaging.

Comparing the dry cathodes it can be observed that the cell with high IEC in the cathode performs better (2.1 V *vs.* 2.2 V at 1.5A cm^−2^) and has a lower HFR than the mid IEC cell (210 mΩ cm^−2^*vs.* 250 mΩ cm^−2^ at 1.5 A cm^−2^, Fig. 4b). Curves are the median of two measurements for each variation, resulting in large error bars especially for the mid IEC cell. This significant deviation may be an indication that the mid IEC dry cathode is the least resilient and more dependent on small changes in assembly and sample history. Although the data has to be interpreted with care due to large error bars, this confirms the findings of neutron imaging, where the high IEC cell had better membrane water retention capabilities at high current densities.

The differences in *iR*-free voltage again could be explained by water content. Taking into account that the majority of the gas transport through the ionomer takes place in the water domains, higher water content leads to higher gas permeability.^20^ This relation therefore could explain the increasing mass transport over potentials (at 1.5 A cm^−2^) with decreasing water content. It is to note, that the different IEC could also lead to different ionomer film thicknesses, which would have the same effect. However, taking the wet sample into account, it becomes apparent that the water content does play a major role.

The mid IEC dry cathode also showed the strongest variation in the high frequency resistance over the range of samples measured, possibly indicating a high sensitivity to changes in relative water content.

### Degradation

As a first indication of the stability of the system the CCMs were subjected to a stress test in form of a constant current hold of 1 A cm^−2^ for six hours. The degradation of the CCMs is dominated by mechanical instability of the anode catalyst layer as discussed in previous work.^14^ However, in direct comparison of the cells tested here, a significantly higher degradation rate is observed in dry cathode operation, which is partially mitigated by the application of an ionomer with higher IEC in the cathode catalyst layer. During the first two hours, the overpotential of the wet cathode and high IEC cell dry cathode cells degrade with approximately 45 mV h^−1^, while the mid IEC cell has a degradation rate of almost 75 mV h^−1^. The increased degradation rate under drier conditions can be explained by an elevated hydroxide activity due to a smaller solvation shell. Thus it is in line with current understanding of ionomer degradation.^10,21^

## Conclusions

The goal of this study was to elucidate the water distribution *in situ* in AEMWE cells when operated with a dry cathode. For this purpose, anion exchange membrane water electrolyzer MEAs were fabricated with two different cathode ionomer ion exchange capacities IECs (1.8–2.2 meq. g^−1^ and 2.3–2.6 meq. g^−1^). *In situ* neutron imaging confirmed that dry cathode operation leads to an expected imbalanced water distribution with high water content in the anode and a gradient inside the MEA towards the dry cathode. Further, this imbalance increases with current density, as water on the cathode side is either consumed in the hydrogen evolution reaction or transported to the anode *via* electro-osmotic drag.

**Fig. 5:**
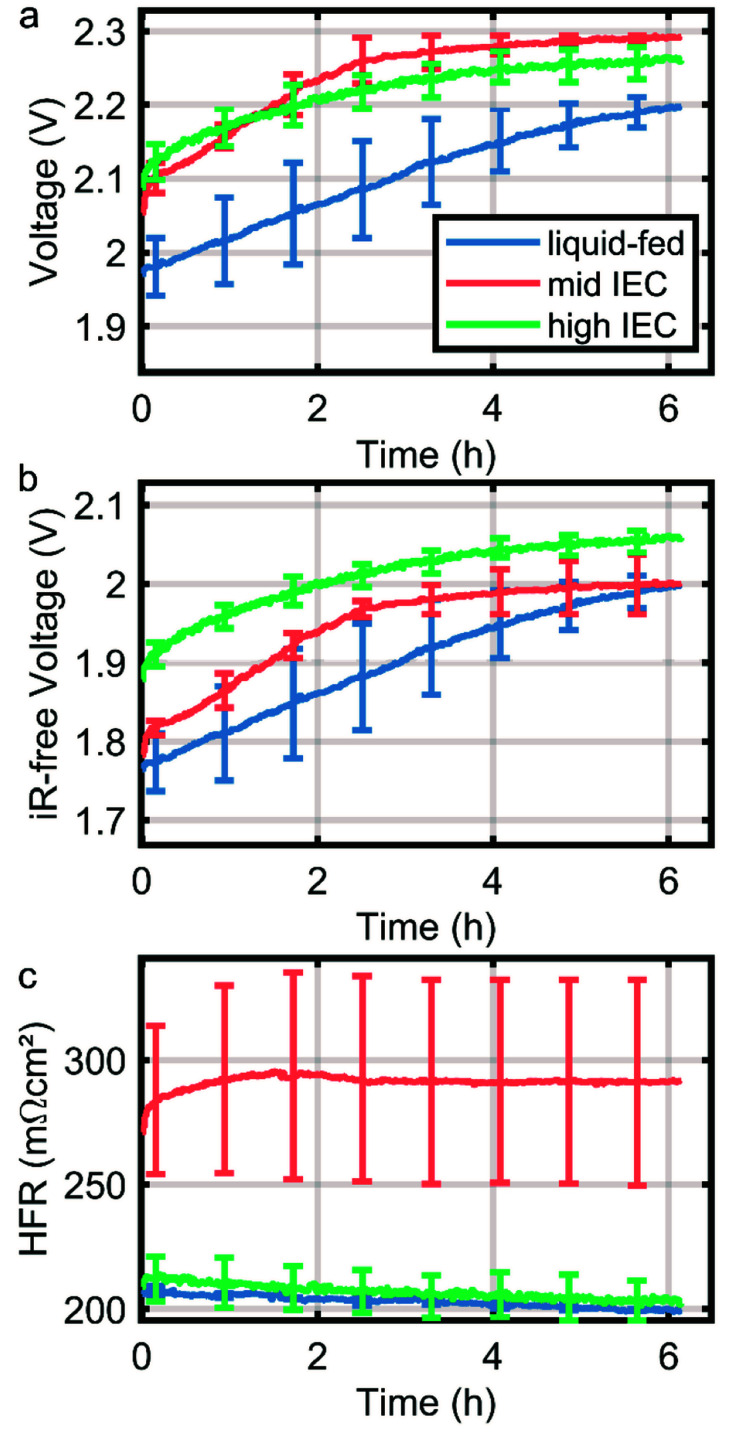
Short-term degradation during 1 A cm^−2^ current hold over up to six hours. Curves are the median of two measurements of each variation and the error bars note the range in which other measurements fall.

Comparing the water distribution of the two cathodes with varying IEC ionomers revealed that a higher IEC ionomer in the cathode catalyst layer lead to better water retention in the MEA compared to the lower IEC over the whole current range. This behavior is expected, as the IEC majorly determines the water content inside an electrode. Polarization data confirms this finding, showing lower overpotentials and membrane resistance for high IEC cells especially at high current densities. The higher IEC further improves the performance stability of the MEA (Fig. 5), which again most probably is linked to an improved water retention and protection of the membrane polymer.

While the high IEC cathode still did not reach the stability and the performance at high current densities of the liquid fed cathode, tuning the IEC inside the cathode offers a powerful way of adjusting the water management inside a dry-cathode AEM water electrolyzer making it a potential alternative to the liquid fed AEM water electrolysis.

## Author contributions

S. K. and S. V. planned the project and mainly drafted this manuscript. J. D. contributed strongly to experiment planning and execution and designed the neutron imaging cell fixture. S. K. K. and S. K. carried out sample fabrication and electrochemical measurements. L. M. assisted with analysis and provided critical feedback on all sections. Y. H. and M. S. conceptualized and performed the neutron radiography measurements together with A. T. and L. H., Y. H. wrote the method section on neutron radiography with contributions from M. S., A. T., L. H. and S. K. All authors critically read and discussed the manuscript.

## Conflicts of interest

There are no conflicts to declare.

## Supplementary Material

RA-012-D2RA03846C-s001
