# Supplementary material for: Water management in anion-exchange membrane water electrolyzers under dry cathode operation
Source: RSC Adv. 2022 Jul 20;12(32):20778–84. doi: 10.1039/d2ra03846c (PMC9297697; doi:10.1039/d2ra03846c)
Supplement: RA-012-D2RA03846C-s001 [file RA-012-D2RA03846C-s001.pdf]

This journal is © The Royal Society of Chemistry 2022

Supplementary Information

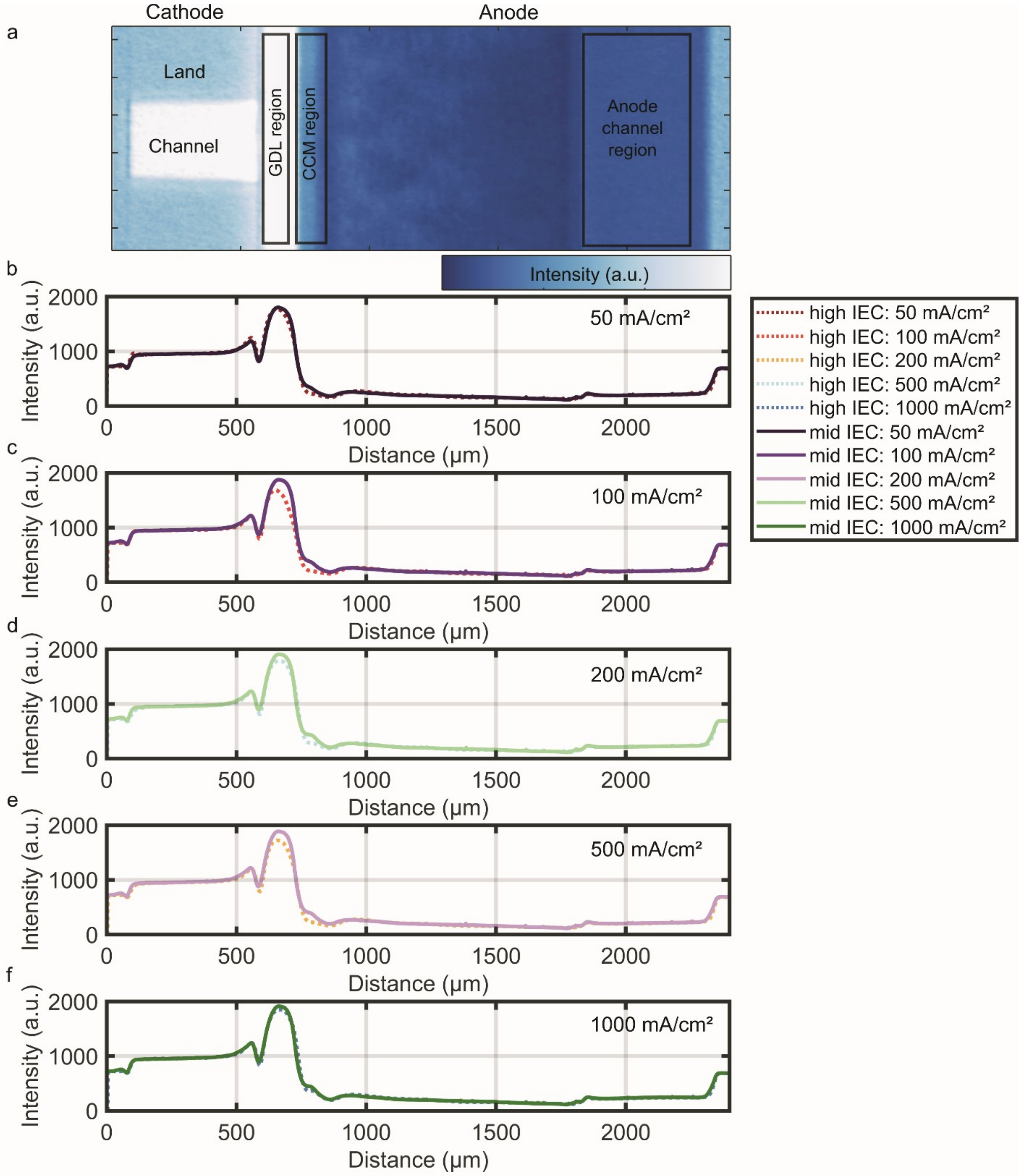

SFig. 1 Neutron image of one channel of the AEM water electrolyzer, b-f Plot profiles of averaged neutron image intensity across one channel of the AEM water electrolyzer for the mid IEC (lines) and high IEC (dotted line) samples at increasing current densities.

The water content of one cathode channel and land estimated from neutron radiography is shown in SFig. 1a to illustrate the relevant regions and distances. SFig. 1b-f show profiles of the intensity of the neutron image across one cathode channel cut horizontally. Within in the metallic ridges of the flow wields and even within the channels on both anode and cathode side all current densities overlap well for the mid and high IEC cell. This

allows a direct comparison of the two cells. In the MEA region a lower intensity is observed for the high IEC cell, especially for 100, 200 and 500 mA/cm². This indicates a higher water content, since water attenuates the neutron signal (see [16] for details).

SFig. 2 shows the transient response of the estimated water content in the cathode GDL and MEA areas, as well as the voltage response of the cell at the applied current densities as well as the HFR extracted from an EIS measurement at the end of each current density point. A significant transient effect can be seen in the neutron transmission, especially for the high IEC cathode binder. This steep incline happens during the first three minutes of measuring and is assumed to result from the water redistribution inside the cell during start up. Before each measurement point the cell was kept at 0 V to allow comparability between the current densities, which results in a steep rise in voltage, which is sometimes seen in the voltage response during the first few minutes in electrochemical data as well. The water distribution response becomes faster and more instantaneous at higher current densities, where a flat response is observed after the first several measurement points. In the lower current

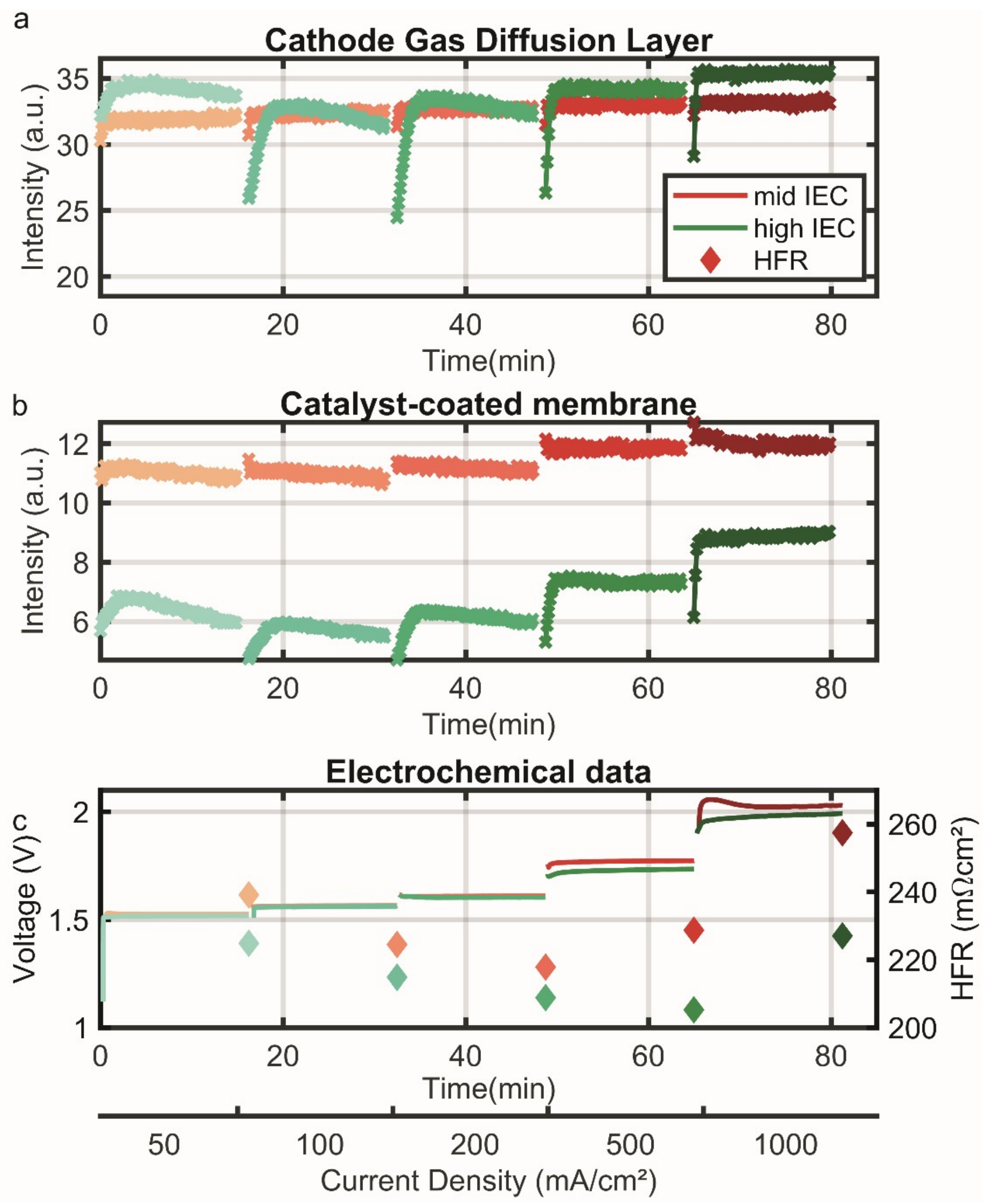

SFig. 2 Transient response of the water content extracted from the respecitve areas in neutron images (a & b) and the measured potential under applied the applied current densities as well as the high-frequency resistance extracted at the end of each measurment (c).

density range of 50-200 mA/cm² the response for the high IEC cell does not even stabilize over the ten minutes of measurement but keeps rising indicating a persisting transient rearrangement of the water or materials in the system.
